# Editorial: Mechanics and regulation of mitotic exit and cytokinesis

**DOI:** 10.3389/fcell.2023.1191987

**Published:** 2023-03-29

**Authors:** Pier Paolo D’Avino, Paola Vagnarelli, Andrew Wilde

**Affiliations:** ^1^ Department of Pathology, University of Cambridge, Cambridge, United Kingdom; ^2^ Department of Life Sciences, College of Health, Medicine, and Life Sciences, Brunel University London, London, United Kingdom; ^3^ Department of Molecular Genetics and Department of Biochemistry, University of Toronto, Toronto, ON, Canada

**Keywords:** abscission, cancer, microtubules, midbody, central spindle, contractile ring, phosphorylation, actomyosin

The process of cell division has fascinated scientists for generations because of its intrinsic beauty and its role in growth, development, and reproduction in all organisms. Cell division controls the faithful segregation of genomic and cytoplasmic materials between two daughter cells and errors during this process have been linked to various human diseases, including cancer (Dominguez-Brauer et al., 2015; Lens and Medema, 2019). This Research Topic of articles in Frontiers of Cell and Developmental Biology focuses on the recent progresses in our understanding of the mechanisms and signalling pathways that regulate exit from mitosis and the separation of the two daughter cells during cytokinesis (D'Avino et al., 2015).

Once the early mitotic events are completed and the spindle assembly checkpoint that monitors proper chromosome-spindle attachments is satisfied, then cells exit mitosis. This process is unidirectional and leads to both the separation of the genomic material but also to the re-establishment of all the cellular compartments that have been dismantled or re-organised to allow mitosis to occur. As this process is very rapid and cell synchronisation difficult to obtain at this specific stage, research on the early stages of mitotic exit has lagged behind. However, recent advances started to shed light on key molecular events that regulate this cell cycle transition (Vagnarelli, 2021). A breakthrough has been the identification of the major protein phosphatases that conduct the reversal of the mitotic wave of phosphorylation; as it has emerged, while kinases govern the early mitosis kingdom, phosphatases reign in the mitotic exit one. Here two reviews cover the recent advances, from a molecular point of view, on how the birth of a new interphase nucleus is controlled. In the first review, Lacroix et al. (Lacroix et al.) focus on the spatial and temporal regulation of Protein Phosphatase 2A (PP2A), how the recognition and specificity for substrates is achieved and how PP2A-B55-dependent dephosphorylation drives mitotic exit. While highlighting the discovery of key substrates for this phosphatase complex important for driving mitotic exit, they also point at the main knowledge gaps such as the understanding of how dephosphorylations events are temporally ordered. In the second review, Archambault et al. (Archambault et al.) focus on the role of de-phosphorylation in the reformation of the nucleus: from the mechanisms involved in clustering the chromosomes together, to direct the deposition of membranes around the chromatin, sealing the membranes and re-assembling the lamina and re-building the nuclear pore complexes. This thorough analysis clearly reveals that several Protein Phosphatase 1 (PP1) enzymes play a central role in the process but also that additional functions of these and other phosphatases will almost surely emerge in the coming years.

Our current understanding of the mechanisms that underpin successful cytokinesis largely stem from the use of a restricted number of model systems. Initial studies often focused on early embryonic divisions in marine organisms (Rappaport, 1961; Rappaport, 1996). As time progressed these were complemented with the rise of more genetically tractable systems and advances in the ability to manipulate tissue culture cells (D'Avino et al., 2015). However, the diversity of systems remained limited. Whilst these models have made an enormous impact on our understanding of the event that drive cytokinesis, it is becoming increasingly clear that they may only offer a small window into the molecular processes driving cell division. Two reviews in this Research Topic bring together observations made in diverse models that highlight the need to investigate cell division in different cells and tissues in order for us to gain a comprehensive understanding of the molecular events that drive all stages of cytokinesis. Ozugergin and Piekny (Ozugergin and Piekny) focus on the mechanistic observations made in different tissue culture systems that highlight subtle differences and the future need to expand our analysis beyond the work horse model of HeLa cells exploited by so many. In contrast, Gerhold et al (Gerhold et al.) focus on the different modes of cell division within the germ line and in particular those where the division process is incomplete. Here they make a clear case of the future need to further study these partial germ line divisions to gain new insight into how the different stages of cytokinesis are regulated. With the advent of new genetic, molecular biology and microscopy tools and techniques that allow these additional models to be exploited, we are on the cusp of an exciting period of cytokinesis research that will reveal a much deeper understanding about the mechanisms driving cell division.

Finally, two articles review our current knowledge of the mechanisms involved in the final separation, or abscission, of the two daughter cells at the end of cytokinesis. After completion of cleavage furrow ingression, the two daughter cells remain connected by an intercellular bridge (IB) which contains an organelle, the midbody, that acts as a platform for the recruitment and regulation of the proteins involved in the final scission event. Andrade and Echard (Andrade and Echard) elegantly and comprehensively review the mechanics and regulation of abscission in animal cells. They initially discuss the role of the tension generated at the IB and how, counterintuitively, high IB tension inhibits abscission. They describe the cellular and molecular components that contribute to IB tension and how they could regulate the activity and assembly of the ESCRT-III proteins, which mediate the final membrane fission event during abscission. In particular, they discuss how cytoskeletal proteins, membrane lipid composition, and membrane structures like caveolae contribute to IB tension and to its release necessary to trigger abscission. In the end, they also discuss whether the mechanisms that regulate abscission in cultured cells apply to all cell types, and how cells could potentially “sense” IB tension. A second review article discuss the role of integrins in cytokinesis and in maintaining genomic integrity (Rani et al.). The authors discuss that integrin-mediated adhesion plays an important role in abscission in human cells by regulating the timing of the recruitment to the midbody of the protein Cep55, which in turn is responsible for the recruitment of ESCRT-III components. This regulation seems to occur through premature degradation of the mitotic kinase Plk1, which phosphorylated Cep55 to prevent its midbody localization until completion of furrow ingression. Interestingly, both articles also discuss how the process of “traction-mediated cytoplasmic fission” or “cytofission”, which is not dependent on ESCRT-III, could represent an ancient mechanism of cell separation at the end of cytokinesis that could still be employed by some cell types in emergency situations to prevent tetraploidy and genomic instability.
